# Arbuscular Mycorrhizal Fungi as Natural Biofertilizers: Let's Benefit from Past Successes

**DOI:** 10.3389/fmicb.2015.01559

**Published:** 2016-01-19

**Authors:** Andrea Berruti, Erica Lumini, Raffaella Balestrini, Valeria Bianciotto

**Affiliations:** Institute for Sustainable Plant Protection - Turin UOS, National Research CouncilTorino, Italy

**Keywords:** arbuscular mycorrhizal fungi (AMF), abiotic and biotic stress, plant nutrition, inoculation, transcriptomics

## Abstract

Arbuscular Mycorrhizal Fungi (AMF) constitute a group of root obligate biotrophs that exchange mutual benefits with about 80% of plants. They are considered natural biofertilizers, since they provide the host with water, nutrients, and pathogen protection, in exchange for photosynthetic products. Thus, AMF are primary biotic soil components which, when missing or impoverished, can lead to a less efficient ecosystem functioning. The process of re-establishing the natural level of AMF richness can represent a valid alternative to conventional fertilization practices, with a view to sustainable agriculture. The main strategy that can be adopted to achieve this goal is the direct re-introduction of AMF propagules (inoculum) into a target soil. Originally, AMF were described to generally lack host- and niche-specificity, and therefore suggested as agriculturally suitable for a wide range of plants and environmental conditions. Unfortunately, the assumptions that have been made and the results that have been obtained so far are often worlds apart. The problem is that success is unpredictable since different plant species vary their response to the same AMF species mix. Many factors can affect the success of inoculation and AMF persistence in soil, including species compatibility with the target environment, the degree of spatial competition with other soil organisms in the target niche and the timing of inoculation. Thus, it is preferable to take these factors into account when “tuning” an inoculum to a target environment in order to avoid failure of the inoculation process. Genomics and transcriptomics have led to a giant step forward in the research field of AMF, with consequent major advances in the current knowledge on the processes involved in their interaction with the host-plant and other soil organisms. The history of AMF applications in controlled and open-field conditions is now long. A review of biofertilization experiments, based on the use of AMF, has here been proposed, focusing on a few important factors that could increase the odds or jeopardize the success of the inoculation process.

## Introduction

Soil microorganisms such as arbuscular mycorrhizal fungi (AMF or AM fungi) represent a key link between plants and soil mineral nutrients. Thus, they are collecting growing interest as natural fertilizers. AMF are obligate symbionts, belonging to the phylum Glomeromycota (Schüßler et al., 2001), which form mutualistic symbioses with about 80% of land plant species, including several agricultural crops. They provide the host plant with mineral nutrients and water, in exchange for photosynthetic products (Smith and Read, 2008). The AMF mycelium that emerges from the root system can acquire nutrients from soil volumes that are inaccessible to roots (Smith et al., 2000). Furthermore, fungal hyphae are much thinner than roots and are therefore able to penetrate smaller pores (Allen, 2011). Carbohydrates and mineral nutrients are then exchanged inside the roots across the interface between the plant and the fungus. AM fungal hyphae exclusively colonize the root cortex and form highly branched structures inside the cells, i.e., arbuscules, which are considered the functional site of nutrient exchange (Balestrini et al., 2015). Thus, AMF can alleviate the limitation in plant growth caused by an inadequate nutrient supply (Nouri et al., 2014). It has recently been suggested that, in natural environments, a non-mycorrhizal condition should be viewed as abnormal for the majority of species (Smith and Smith, 2012), although there is a marked diversity among AM fungal communities belowground, depending on plant species diversity, soil type, and season, or a combination of these factors (Smith and Smith, 2012).

In addition to an improved nutritional supply, AM interactions provide other benefits to plants, such as improved drought and salinity tolerance (Augé, 2001, 2004; Porcel et al., 2011; Augé et al., 2015) and disease resistance (Pozo and Azcón-Aguilar, 2007). Although several works have been devoted to studying the influence of AM symbiosis on the plant response to abiotic stress (such as drought, salinity, and flooding) in the last few years, the mechanisms responsible for the increased plant tolerance to stress have yet to be fully elucidated (Augé, 2001; Ruiz-Lozano, 2003; Ruiz-Lozano and Aroca, 2010; Bárzana et al., 2012, 2015; Ruiz-Lozano et al., 2012; Calvo-Polanco et al., 2014; Saia et al., 2014; Augé et al., 2015; Sánchez-Romera et al., 2015). Metals such as Fe, Cu and Zn play essential roles in several subcellular compartments, but they constitute a highly reactive group of elements that are toxic at high concentrations (Tamayo et al., 2014, and references therein). AM fungi are known to alleviate heavy metal toxicity in the host plants and to tolerate high metal concentrations in the soil (Göhre and Paszkowski, 2006; Lingua et al., 2008; Cornejo et al., 2013; Tamayo et al., 2014; Meier et al., 2015). Metal transporters play a central role in heavy metal homeostasis. A Zn transporter has been identified in *Glomus intraradices* (*GintZnT1*) (González-Guerrero et al., 2005) and, more recently, through a genome-wide analysis of the recently published *Rhizophagus irregularis* (formely *Glomus intraradices* DAOM-197198) genome (Tisserant et al., 2013), several putative genes coding for Cu, Fe, and Zn transporters have been identified (Tamayo et al., 2014). The next steps will involve the functional characterization of these transporters and the identification of their roles in the symbiosis.

Furthermore, AM fungi can also have a direct effect on the ecosystem, as they improve the soil structure and aggregation (Rillig and Mummey, 2006; Leifheit et al., 2014, 2015; Rillig et al., 2015) and drive the structure of plant communities and productivity (van der Heijden et al., 1998). The influence of AM symbiosis on greenhouse gas (GHG) emissions has recently been investigated (Bender et al., 2014; Lazcano et al., 2014). Bender et al. (2014) have demonstrated that AM fungi contribute to reducing emissions of N_2_O, which is an important greenhouse gas, thus suggesting that they could play a role in the mitigation of climate change. AM fungi could regulate N_2_O emissions by enhancing plant N uptake and assimilation, which results in the reduction of soluble N in the soil, and, consequently, in a limitation of denitrification (Bender et al., 2014). Furthermore, the correlation between the key genes involved in N_2_O production (*nirK*) and consumption (*nosZ*) and AM fungal abundance suggests that the regulation of N_2_O emissions is caused by changes induced by AM fungi in the soil microbial biomass and in the community composition. AM fungi could have an indirect influence on GHG emissions, and also change the physical conditions of soil, i.e., moisture, aggregation, and aeration, all of which influence the production and transport of GHG in soil. Lazcano et al. (2014) have reported that AM symbiosis helps to regulate N_2_O emissions at high soil moisture levels and suggested that the control of N_2_O emissions by AM plants could be driven by a higher use of soil water rather than by increased N uptake.

Thus, AM fungi are primary biotic soil components which, when missing or impoverished, e.g., due to anthropic input, can lead to a less efficient ecosystem functioning. The process of re-establishing the natural level of AMF richness can represent a promising alternative to conventional fertilization practices, with a view to sustainable agriculture, a key target for growers facing the global recession and having to deal with a more environmentally aware clientele. The main strategy adopted to achieve this goal is the direct re-introduction of AMF propagules (inoculum) into a target soil. However, the exploitation of these fungi in applicative programs requires the knowledge of how AMF adapt and react to the target ecosystem and soil management and of the events that lead to the establishment of a functional symbiosis, including the mechanisms involved in nutrient transfer. In this review, after a brief mention of the most recent results on the nutritional aspects of AM symbiosis and a quick overview of the challenges involved in AMF inoculum production, examples of AM fungi application, in controlled and open-field conditions, are reported and discussed, with particular attention being paid to identifying the factors that led to successful outcomes in the biofertilization experiments.

## New insights into mineral nutrition in AM symbiosis

Since the characterization of a high-affinity phosphate transporter (PT) in an AM fungus (Harrison and van Buuren, 1995), the nutritional aspects of AM symbiosis have been studied extensively from both a physiological and a molecular perspective (Harrison et al., 2002; Paszkowski et al., 2002; Nagy et al., 2005; Bucher, 2007; Smith and Smith, 2011, 2012). AM fungi are capable of significantly improving plant mineral nutrient acquisition, mainly in low-nutrient conditions, and it has clearly been demonstrated that plants possess a symbiotic Pi uptake pathway (Harrison et al., 2002; Paszkowski et al., 2002; Nagy et al., 2005; Bucher, 2007; Smith and Smith, 2011). Radiotracer experiments have made it possible to verify the relative amount of Pi that enters a plant *via* the AM fungus and directly through the root transport system, and have revealed that the fungus can transfer the Pi to the plant even without an evident growth effect (Pearson and Jakobsen, 1993; Smith et al., 2003, 2004). It is well-known that AM symbiosis specifically induces the expression of plant Pi transporters (Harrison et al., 2002; Paszkowski et al., 2002; Nagy et al., 2005; Xie et al., 2013; Walder et al., 2015). In addition to the increase in plant Pi acquisition, a role in regulating arbuscule morphogenesis and in maintaining symbiosis has been demonstrated (Javot et al., 2007, 2011; Yang et al., 2012; Xie et al., 2013). Recently, the expression of the *Medicago truncatula* and *Lotus japonicus* AM-induced Pi transporters *MtPT4* and *LjPT4* has also been found in the root tips of non-colonized plants, and a role of *PT4* genes as components of the Pi-sensing machinery in root tips has been suggested (Volpe et al., 2015). Three PT genes in tomato are up-regulated in AM-colonized roots (*LePT3, LePT4, LePT5*) (Nagy et al., 2005), and the functional characterization of LePT4 has been reported, thus showing its pivotal role during symbiosis (Xu et al., 2007). Evidence of the role of AM symbiosis in the transfer of several mineral nutrients has been obtained in studies on several plant species (Casieri et al., 2013, for a review on the “transportome” in AM symbiosis; Paszkowski et al., 2002; Nagy et al., 2005; Guether et al., 2009a; Hogekamp et al., 2011). In addition to the Pi transporters specifically involved in the uptake from the arbuscules (Harrison et al., 2002; Paszkowski et al., 2002; Nagy et al., 2005), mycorrhiza-inducible ammonium transporters (AMT) have also been identified (Gomez et al., 2009; Guether et al., 2009b; Kobae et al., 2010; Koegel et al., 2013). The periarbuscular membrane, which is the plant-derived membrane that envelops the arbuscule, is considered the site in which the last stages of the symbiotic mineral nutrient transfer occur: plant transporters located in this membrane can capture mineral nutrients from the periarbuscular apoplast and transfer them to the cortical cell (Javot et al., 2011; Bapaume and Reinhardt, 2012). AMTs have been located in the periarbuscular membrane of soybean and *Medicago*, as previously demonstrated for the Pi transporter MtPT4 in *Medicago truncatula* (Harrison et al., 2002), thus suggesting a role in ammonium transport to the cortical cells (Kobae et al., 2010; Breuillin-Sessoms et al., 2015). Taken together, the analyses on Pi and ammonium transporter Medicago mutants have demonstrated that the Pi and AMT symbiotic transporters (i.e., PT4 and AMT2;3) have an influence on the arbuscule lifespan (Javot et al., 2007; Breuillin-Sessoms et al., 2015). It has been speculated that the transport of Pi or ammonium through these transporters not only delivers nutrients to the root cells but also triggers signaling that enables conditions for arbuscule maintenance (Breuillin-Sessoms et al., 2015).

In addition to Pi and N, sulfur (S) can also be transferred to plants through AM fungi (Allen and Shachar-Hill, 2009; Sieh et al., 2013). In fact, AM symbiosis improves the S nutritional status of the host plant, affecting the expression of plant sulfate transporters (Casieri et al., 2012; Giovannetti et al., 2014). In this context, the application of laser microdissection technology has allowed the expression of Pi and NH_4_ tansporters in arbusculated cells to be verified (Balestrini et al., 2007; Gomez et al., 2009; Guether et al., 2009b) and, more recently, a sulfate transporter specifically involved in the uptake from the arbuscules has also been identified (Giovannetti et al., 2014). On the fungal side, a phosphate (Balestrini et al., 2007; Tisserant et al., 2012; Fiorilli et al., 2013) and an ammonium transporter (Pérez-Tienda et al., 2011) have been found to be expressed in arbuscules, which would seem to suggest that the fungus may reabsorb nutrients released at the periarbuscular interface, thus exerting a control over the amount of nutrients delivered to the host (Balestrini et al., 2007). In spite of the importance of potassium (K^+^) for the plant cell machinery, the contribution of AM symbiosis to plant K^+^ nutrition has rarely been studied (Garcia and Zimmermann, 2014). This element is very abundant in soil, but its availability is very low due to its strong mineral adsorption. The accumulation of this element in AM fungi has been reported in spores (Pallon et al., 2007), hyphae (Olsson et al., 2008), and vesicles (Olsson et al., 2011). Furthermore, the up-regulation of a plant K^+^ transporter has been reported in mycorrhizal *Lotus japonicus* roots (Guether et al., 2009a). Interestingly, the K^+^ derived from AM symbiosis can be correlated to an improved plant tolerance to abiotic stress, e.g., salinity and drought (Garcia and Zimmermann, 2014 and references therein). Recently, two meta-analysis studies, focused on the contribution of AM symbiosis to different micronutrient concentrations in crops, have been published (Lehmann et al., 2014; Lehmann and Rillig, 2015). AM fungi could be used as a sustainable tool to improve micronutrient concentrations in crops, as an alternative to, or in addition to genetic and agronomic biofortification (i.e., the increase in the concentrations and/or bioavailability of mineral elements in plant products). Besides crop productivity, AM symbiosis, due to its role in plant nutrition, could also have a positive impact on crop quality, thanks to the enrichment in both macro and micronutrients (Antunes et al., 2012; Hart and Forsythe, 2012; Pellegrino and Bedini, 2014). Focusing on Zn, Lehmann et al. (2014) concluded that AM symbiosis positively affected the Zn concentration in various crop plant tissues under distinct environmental conditions. Soil texture, pH, and soil nutrient concentration (i.e., Zn and Pi deficiency) have in fact an influence on the AM fungus-mediated Zn content in different plant tissues (Lehmann et al., 2014). Moving attention to copper (Cu), iron (Fe), and manganese (Mn), the study by Lehmann and Rillig (2015) has shown that there is a positive impact of AM symbiosis on crop plant Cu and, for intermediate experimental duration (lasting 56–112 days), Fe nutrition, while a benefit for plant Mn nutrition has only been observed in herbs. Pellegrino and Bedini (2014) have demonstrated that AM fungal field inoculation could be an effective tool to improve the cultivation of chickpea as it can improve productivity, but also the grain nutritional content in protein, Fe and Zn.

Interestingly, it has been proposed that the nutrient-dependent regulation of AM colonization provides an important feedback mechanism for plants to promote or limit fungal colonization according to their needs (Nouri et al., 2014). It has already been demonstrated that phosphorous availability represents an environmental factor that can disturb the symbiotic interaction of AM. In fact, the suppression of AM colonization due to high Pi levels has been reported in several experiments (Breuillin et al., 2010; Balzergue et al., 2013; Bonneau et al., 2013). Recently, in order to evaluate which nutrients, together with phosphorous, influence AM development, several elements have been tested to verify the inhibitory effects on AM colonization, using *Petunia hybrida* and *R. irregularis* as AM systems (Nouri et al., 2014). The results have shown that Pi, in agreement with previous data on the same AM system (Breuillin et al., 2010), and nitrate can potentially exert negative regulation on AM, while sulfate, Mg^2+^, Ca^2+^, and Fe^3+^ have no effect. Furthermore, the starvation of several nutrients, in particular of nitrate, has been shown to reverse the inhibitory effect of Pi on AMF, thus suggesting that nutrient starvation triggers a dominant AM-promoting signal that counteracts the effects of high Pi levels (Nouri et al., 2014). However, considering recent evidence on nutrient exchange in AM symbiosis, Walder and van der Heijden (2015) have reported that the cooperation in AM interactions is related to the partners involved in the symbiosis, and depends on several factors, including environmental conditions, acquisition of surplus resources and functional diversity.

## Challenges related to AMF inoculum production and application

The need to benefit from AMF as a biofertilizer, with a view to sustainable agriculture, is becoming increasingly urgent since the appropriate management of these symbiotic fungi could potentially decrease the use of agrochemicals. The main strategy adopted to achieve this goal is the inoculation of AMF propagules (inoculum) into a target soil. Unfortunately, AMF are obligate symbionts and cannot be cultivated in pure cultures, away from their host plants. This constraining feature makes the large-scale production of AMF inocula very challenging and complex. There are three main types of AMF inocula. First, soil from the root zone of a plant hosting AMF can be used as inoculum as it normally contains colonized root fragments, AMF spores, and hyphae. However, unless precise information about the propagule abundance, diversity, and infectivity are available, soil inocula can be unreliable and carry the possible risk of transferring weed seeds and pathogens. Spores extracted from soil can instead be used as starters for crude inoculum production. Crude inoculum can be obtained after a known isolate of AMF and a host trap plant (i.e., a plant that can be massively colonized by many AMF species) are grown together in an inert medium optimized for AMF propagation. This is the most commonly used type of inoculum for large-scale crop inoculation as it usually contains a more concentrated set of the same kind of propagules found in soil inocula. Finally, infected root fragments alone of a known AMF host that have been separated from a trap plant culture can also serve as a source of inoculum.

The production of AMF crude inoculum on a large-scale remains very challenging even though new methods for massive production (IJdo et al., 2011) and seed coating technology (Vosátka et al., 2013) have been developed in recent years (van der Heijden et al., 2015). The main obstacle to the production of an AMF inoculum lies in the obligate symbiotic behavior of AMF, that is, their need to have a host plant for growth and completion of their life cycles. This means that the propagation step must include a phase of cultivation with the host plant that is usually time and space-demanding. As a consequence, the setting up of AMF reference collections also requires methodologies that are rather different and more binding than those used for other microbial collections. Moreover, the absence of a prompt method for assessing whether and to what extent the host plant is colonized by AMF also contributes to making AMF agricultural usability challenging. The management of the high amount of inoculum necessary for large-scale application is also a demanding process. However, AMF inoculation is carried out more easily for plant production systems that involve a transplant stage, since smaller amounts of inoculum are needed.

At a first glance, carrying out an open-field, extensive inoculation treatment could seem technically impractical and economically prohibitive. However, once AMF biodiversity is restored and well-established, and if an AMF-friendly management, such as fall cover cropping (Lehman et al., 2012) and conservation tillage (Säle et al., 2015) is put in place, the AMF community will persist. If no detrimental practices are carried out before and after cultivation, it is known that the biodiverse mycorrhizal hyphal network will remain unaltered and infective in the future. As an alternative to large-scale inoculation, a small-scale approach is also feasible. Taking inspiration from the idea of creating the so-called “fertility islands” (Allen, 1987), AMF inoculation could be limited to small portions of a field, and this would gradually lead to the establishment of a healthy AMF mycelial network, but with reduced costs. This technique would be particularly indicated when the AMF inoculation is aimed at assisting the revegetation of a degraded land, since inoculated fertility islands likely allow native plant species to recover the nutrient impoverished land faster.

Hence, AMF restoration only represents an initial cost that, if the persistence of AM fungi is favored in the soil, could be prorated over the years. As already demonstrated (Gulati and Cummings, 2008; Barr, 2010), AMF inoculation can be economically profitable, in comparison to conventional fertilization, providing substantial savings for growers and for degraded land recovery projects. In order to provide further data to assess AMF inoculation attractiveness, it is important that the end-users should also cultivate an uninoculated portion of their crop, so as to be able to evaluate the cost-effectiveness and beneficial effects on plant fitness due to AMF (Dalpé and Monreal, 2004).

The global economic crisis is now forcing growers to try to understand the potential of sustainable agricultural systems, and of reducing the input of phosphorus using AMF inocula. Unfortunately, solid inoculation practices have yet to be implemented, and applied research focused on defining the best inoculum formulation strategies (Verbruggen et al., 2013) should be encouraged. The potential of AMF has drawn the attention of the commercial sector, and several companies nowadays produce and sell AMF-based inocula. The general tendency is to formulate inocula with only a few AMF species as components. Some manufacturers have chosen the single formulation approach, but others produce different products that are supposedly targeted for end-users who are willing to apply the formulation to a range of environmental conditions and host-plant groups. The few species that are used can easily be routinely propagated and are normally generalist, as they are found in association with a large variety of host plants in different biomes. Although commercial inocula are often advertised as suitable for a wide range of plants and environmental conditions, the real benefits are not always positive (Corkidi et al., 2004; Faye et al., 2013). For this reason, in order to promote AMF inoculum market development and improvement, scientists should strengthen the link between research and companies and introduce a series of “best practices” that could be adopted to solve issues related to the functioning of commercial inocula. One of these issues arises from the need to control the biological composition of a product, for example, due to the possible presence of pathogens and weeds (Douds et al., 2005; Tarbell and Koske, 2007), but above all to the need to assess its purity in terms of AMF composition. In fact, the species list declared in a commercial inoculum label does not always correspond accurately to the actual inoculum composition (Berruti et al., 2013a). In addition, being obligate symbionts, AMF inocula are mostly produced using a containerized-culture, either in greenhouses, growth chambers, or in fields, and, as a result, cannot possibly be free from external microorganisms. Owing to the increased awareness of the risk of pathogens, many concerned manufacturers are now applying agrochemicals in order to avoid contamination of their product (Douds et al., 2005). In order to reduce pathogen carry-over, it is possible to opt not to include host root residues in the inoculum while, as an alternative, the incorporated root fragments can be surface sterilized without jeopardizing the viability of the AMF propagules (Mohammad and Khan, 2002). Over the last few decades, several techniques have been applied to molecularly characterize entire AMF communities in complex matrices, such as soil (Hempel et al., 2007; Lumini et al., 2010; Borriello et al., 2012; Davison et al., 2012) and AMF inocula (Berruti et al., 2013a,b). These methodologies also allow the inoculated AMF to be monitored inside the host plant during the cultivation cycle (Alkan et al., 2006; Farmer et al., 2007; Pellegrino et al., 2012; Thonar et al., 2012; Werner and Kiers, 2015). High-throughput next generation sequencing (NGS) potentially offers the most powerful and sensitive technique to trace the introduced fungus, both temporally and spatially. This set of techniques also makes it possible to verify whether the inoculated AMF favor significant levels of colonization, although this may not necessarily be important if the effects on crop production and quality are indirect via the resident AMF community (Rodriguez and Sanders, 2015). Finally, NGS also leads to the understanding of how the introduced AMF interact and coexist with the local AMF community (Rodriguez and Sanders, 2015).

## Lessons from past successes and failures of AMF inoculation

**Figure 1:**
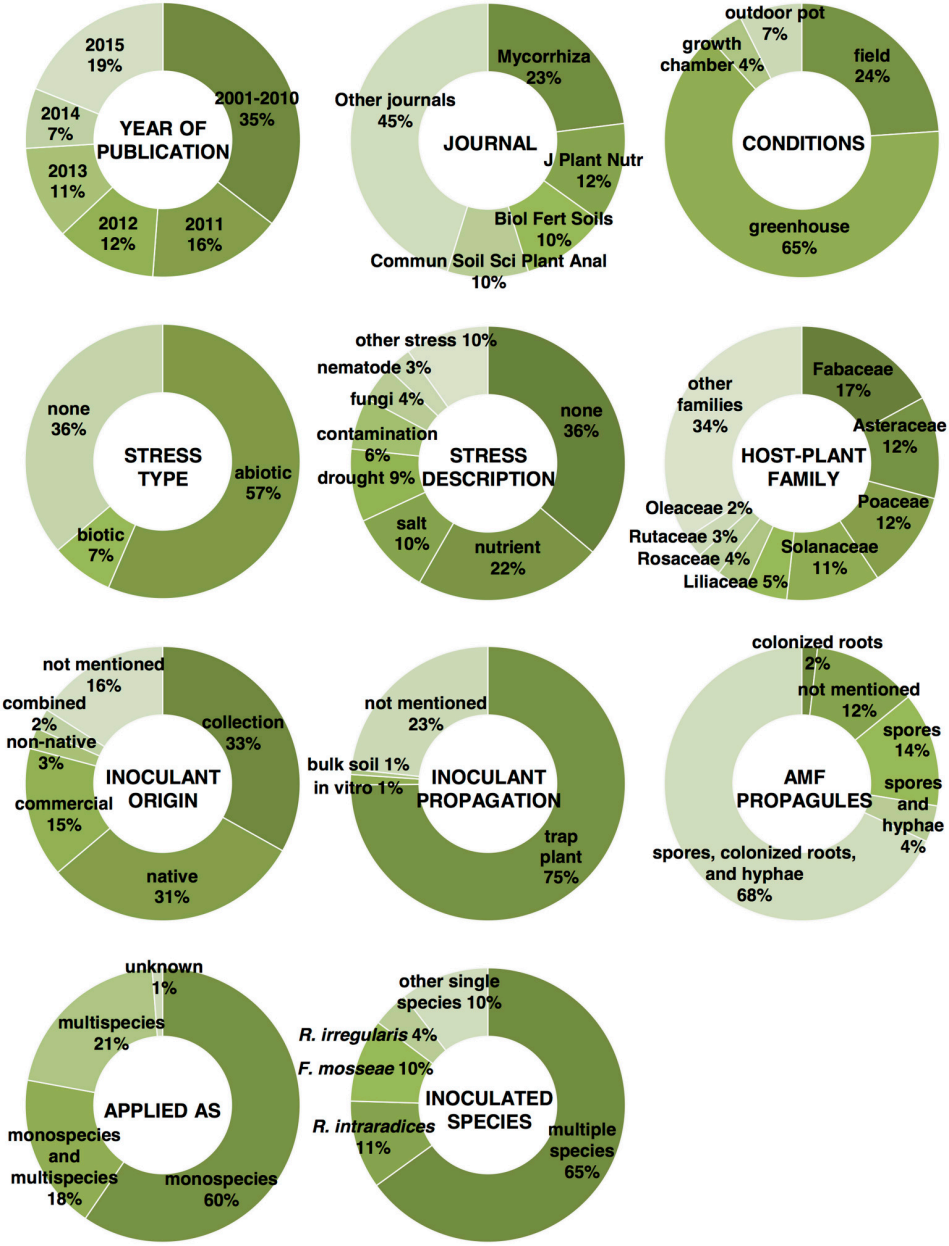
**Percentage of reviewed articles according to the year of publication and to the scientific journal, and percentage of experiments illustrated in the reviewed articles according to the cultivation conditions, stress type, and description, mycorrhizal host-plant family, origin of the AMF inoculants, method of AMF propagation used prior to inoculation, type of AMF propagule, method of application, and inoculated AMF species**.

In order to capitalize the effort that has been made in the past years by researchers in an attempt to deliver a sustainable cropping system based on AMF inoculation, a large number of studies published over the last 15 years have been reviewed in detail in this review. In other words, 127 studies (Supplementary Material S1) were randomly selected from an article list obtained from a search on Google Scholar using the keywords “arbuscular mycorrhizal fungi” and “inoculation,” with a publication date from 2001 to 2015. Overall, articles from 47 different scientific journals have been considered in this review. The most important information and factors featured in the reviewed articles are shown in Figure 1. Most of the studies were published in 2015 and in the 5 year window from 2011 to 2015, thus suggesting that researchers have been dedicating a great deal of effort into trying to implement agriculture with the potential advantages conferred by AMF. These 127 articles deal with 164 inoculation experiments aimed at determining the effect of AM fungi inoculation in different conditions on 43 plant families (mainly Fabaceae, Asteraceae, Poaceae, and Solanaceae) often subjected to abiotic stress. Biotic stress has received much less attention, and many experiments have not included any stress application. This could corroborate the fact that AMF-mediated pathogen protection is still poorly studied and that this aspect deserves more attention. The characteristics of these studies have been described in a table (Supplementary Material S2), in which the main factors involved in each experiment are categorized and the main results that have been obtained are schematically outlined. This table includes information on the experimental condition in which the inoculation study was carried out (field, outdoor pot, greenhouse, or growth chamber), the stress type (biotic or abiotic) against which AMF application was investigated, the host-plant species and family, the inoculant origin, the used inoculum propagation method, the type of AMF propagule applied, the method of application (monospecies, multispecies, or both) and the tested AMF species list. The main results of these inoculation studies are also reported. The results section of the table focuses on whether there was a significantly positive effect (α = 0.05, regardless of which statistical test was applied) or not of the inoculation treatment vs. the respective non-inoculated control. In particular, the table reports six parameters that were considered to assess the AMF inoculation effect in most of the experiments considered in this review. These parameters are the root fungal colonization gain, root and shoot biomass increase, yield and plant nutrition improvement, and plant resistance to a given pathogen. Unfortunately, not all of the studies provided measurement results and statistical analyses for all of the six parameters that were chosen to describe the effects of AM fungi on the plant, but this was to be expected.

Overall, AMF inoculation has appeared to be highly positive for plant development and production in the experiments considered in this review (Supplementary Material S2). A significant root colonization gain, comparing to non-inoculated controls, was registered in 93.8% of the 130 experiments that provided this measurement. Root and shoot biomass was significantly increased by inoculation in 73.6 and 80.8% of 91 and 146 experiments, respectively. Yield and plant nutrition were improved by inoculation in 84 and 92% of 81 and 112 experiments, respectively.

In an effort to define whether some of the most important factors considered in the reviewed studies have the potential to determine the success or the failure of inoculated AMF on plant productivity, the proportions of experiments showing a significant increase in colonization, biomass, yield, and nutrition following AMF inoculation was calculated for three important factors (Supplementary Material S3), i.e., the experimental condition (levels = greenhouse or open-field), the inoculant origin (levels = native origin or other origin), and the method of application (levels = as monospecies inoculum or multispecies inoculum). In order to statistically support the data interpretation, a 2-sample *z*-test was performed in which the percentage values of the two factor levels were compared. In addition, asymptotic confidence intervals were calculated for each percentage value. Providing sample proportions (the percentage of experiments resulting in a significant increase in a given parameter divided by 100) and sample sizes (the number of experiments considered for the calculation of the percentage value), the *z*-test is able to return a *p*-value that can be used to either accept or reject the null hypothesis that the sample proportions are equal.

### Inoculation success in greenhouse and open-field conditions

**Figure 2:**
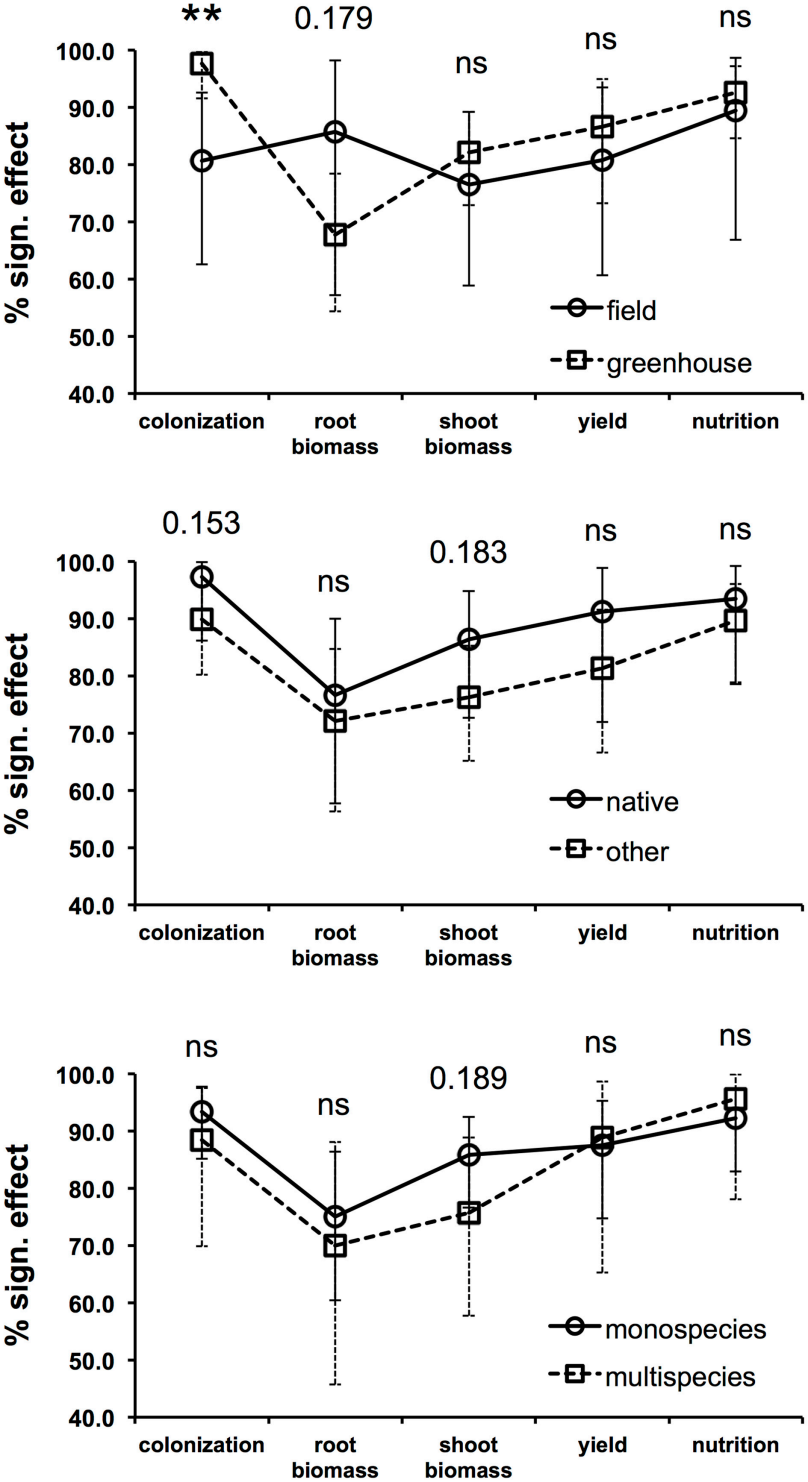
**Percentage of experiments showing significant increases in fungal colonization, root biomass, shoot biomass, yield, and plant nutrition comparing field and greenhouse conditions (upper graph), inoculation with native AMF and AMF of different origin (center graph), and inoculation with only one species and multiple species at the same time (lower graph)**. The statistical significance of the difference between the two proportions (percentage value divided by 100) calculated for each factor (i.e., cultivation condition, inoculant origin, and method of application) was computed with a series of 2-sample *z*-tests. The *z*-test *p*-value is reported for each of the five parameters in each graph (^**^p < 0.01; ns = *p* > 0.20). The asymptotic confidence intervals are reported for each percentage value.

Most of the experiments (65%) were carried out in greenhouses, while 24% were conducted in open-field conditions (Figure 1). As expected, the fungal colonization gain in inoculated plants, compared to non-inoculated controls, was significantly more frequent in the greenhouses than in the open-field conditions (*z*-test *p* < 0.01, Figure 2). This is most likely due to the fact that the non-inoculated control portion of a field often contains AM fungal propagules, while control pots in greenhouses are usually filled with sterilized substrates that are free of AM fungal propagules or highly reduced in AMF diversity. Interestingly, it has been observed that the root biomass benefits more from inoculation in field conditions than in greenhouse conditions, as can be seen from the results of a *z*-test *p*-value in the near-significant range (0.179). This is probably due to the fact that containerized roots stop growing because of constraints imposed by pot boundaries at a certain point in time during cultivation. In addition, the inoculated plants might sometimes be less prone to invest in root growth in pots. This could be due to the fact that containerized inoculated plants are likely to rely massively on fungal-mediated uptake (Smith et al., 2011) and can reach a maximum level of exploration of the substrate sooner than non-inoculated plants, without increasing the root biomass. Conversely, the effectiveness of AMF inoculation on shoot biomass, yield, and plant nutrition does not seem to be affected by the experimental conditions, and has been shown to be equally successful in greenhouse and open-field conditions.

### Inoculum propagation method and source

Among the reviewed studies, it has been found that the most widespread method for AMF propagation prior to inoculation is by using trap plants (75%), and, interestingly, only marginal use is made of other methods. A few other alternatives to the use of potted trap plants are in fact available. Soilless culture systems, such as aeroponics and hydroponics, lead to the production of pure clean spores and the maximization of growing conditions for the host plant (IJdo et al., 2011), and could soon reach massive use for large-scale production. The root-organ monoxenic culture is another method that allows the successful large-scale propagation of AMF that can be used directly as an inoculum. Unfortunately, the protocol has only been implemented for a reduced number of AM fungal species. The method consists in culturing inoculated excised roots (the so-called hairy roots) that have acquired the ability to proliferate without growing any epigeous portion, after transformation with *Agrobacterium rhizogenes* (Bécard and Fortin, 1988), which is a soil-borne bacterium containing the Ri (root-inducing) plasmid. A massive number of spores, mycelium, and colonized roots are obtained from one Petri dish in just a few months (Declerck et al., 1998). As AMF can use a number of different types of propagules in order to grow and colonize new roots with different degrees of efficiency (Klironomos and Hart, 2002), the choice of the inoculum source (described above) is a factor of primary importance for a successful colonization. Components of the extraradical and intraradical phase of AM fungi include spores, mycelium pieces fragmented from the belowground hyphal network, and several structures inside both living and dead root fragments. Intraradical vesicles, in particular, have been shown to be a primary source of regrowth for certain AM fungal species (Biermann and Linderman, 1983). Various AM fungal taxonomical ranks differ in their ability to propagate from a given propagule. Propagation through mycelial fragmentation seems more important for species of the Glomeraceae family, whereas spore germination is the preferential type of propagation for members of other families (e.g., Gigasporaceae, Acaulosporaceae, and Scutellosporaceae; Brundrett et al., 1999). Hence, when wishing to apply a multispecies inoculum, the most eligible and user-friendly solution, since propagation via trap plant is the most commonly used method, is to sieve the substrate and finely chop the roots of the trap plant in order to retrieve all the different types of fungal propagules (crude inoculum). This solution was used in 68% of the reviewed experiments. Spores alone or mixed with hyphae were used in 14 and 4% of the cases, respectively. Mycorrhizal root fragments alone (root inoculum) were used in 2% of the cases.

### Origin of the fungal inoculant

Another important factor involved in the success of the inoculation process is the choice of the AM fungal inoculants. The applied inoculants mostly came from culture collections or were isolated from the same types of soils used in the experiment. A moderate number of experiments featured the use of commercially available AMF-based inocula. Some AMF species are commonly recognized to be more stress tolerant than others, and are usually found in stressed and polluted soils (Leyval et al., 2002; Hildebrandt et al., 2007). Native AMF from areas affected by osmotic stress can potentially cope with salt stress in a more efficient way than other fungi (Ruiz-Lozano and Azcón, 2000). Thus, it is preferable to take this into account when “tuning” an inoculum to a particular kind of degraded/stressed soil and/or in order to avoid failure of the revegetation process (Vosátka et al., 1999; Oliveira et al., 2005). Overall, the reviewed studies point out the higher efficiency of native AMF. For example, indigenous AM fungi resulted in a better plant protection against root-knot nematode (Affokpon et al., 2011), higher growth in Mn contaminated soil (Briccoli Bati et al., 2015), and in a higher shoot biomass in highly calcareous soil (Labidi et al., 2015), than commercial inoculants. In addition, Estrada et al. (2013) demonstrated how, under saline stress, plants inoculated with native AMF had a higher shoot dry weight, efficiency of photosystem II, stomatal conductance, and accumulation of glutathione than those inoculated with AMF from culture collections. As a whole, the success of AMF application is always more frequent when native species are inoculated, although never in the *z*-test significance range (Figure 2). However, the more frequent occurrence of root colonization gain and shoot biomass increase in response to inoculation with native species are supported by the lowest *z*-test *p*-values, both of which are within the near-significance range (0.153 and 0.183, respectively). Most manufacturers advertise their commercial inocula by pointing out their suitability for a wide range of plants and environmental conditions. Unfortunately, the promises made about these products and the obtained results are sometimes far apart. Examples of ineffective or badly formulated inocula can be found in the literature (Corkidi et al., 2004; Garmendia and Mangas, 2014). For example, Corkidi et al. (2004) described an experiment in which commercial inocula that did not promote mycorrhizal colonization were the only ones that were able to improve the growth response of potted corn plants. Hence, the authors hypothesized the presence of other growth promoting additives in the tested inocula. Similarly, Garmendia and Mangas (2014) attributed the positive effect on lettuce growth and nutrition to the high mineral content included in a commercial inoculum. Optimal benefits are therefore more likely to be obtained from inoculation after a careful selection of the favorable host/niche/fungus combinations.

### Composition of the inoculum

The current general trend is to try one or more species of AM fungi for individual inoculation (monospecies inoculum), as seen in 60% of the reviewed experiments. Single species inoculation experiments tend to be more successful for a shoot biomass increase (*z*-test *p* = 0.189, Figure 2) than inoculation experiments with more than one species applied at the same time. Accordingly, Gosling et al. (2015), after assessing there was no beneficial effect on plant growth after inoculating diverse communities of AM fungi with functionally different traits, argued that when the host plant is exposed to a single factor, such as during greenhouse experiments, fewer fungal species able to alleviate that stress are likely to provide maximal benefit to the host, while a more diverse community would be required under multiple stress field conditions. Another greenhouse study has suggested that the composition rather than the diversity of species within a partnership could be more influential in determining how species function (Wagg et al., 2015). Many experiments have been limited to the single inoculation of one of the following three species: *Rhizophagus intraradices, Funneliformis mosseae*, and *R. irregularis* (Figure 1). These are very generalist symbionts that can colonize a large variety of host plants, survive long-term storage, are geographically distributed all over the world (Öpik et al., 2010), and can be easily and massively propagated. The aforementioned characteristics have made these species suitable for premium inoculum components. Several studies have highlighted that different isolates within the same species, rather than different species, can cause larger variations in plant response (Munkvold et al., 2004; Gai et al., 2006; Angelard et al., 2010). This suggests that the widespread use of single AM fungal species, such as *R. intraradices, R. irregularis*, and *F. mosseae*, in inoculation trials should not be considered a flaw as these species can contain considerable functional heterogeneity. In this context, in the presence of the *R. irregularis* reference genome (Tisserant et al., 2013; Lin et al., 2014), the partial genome re-sequencing of multiple isolates from different geographic origins will pave the way toward the study of the functional implication of genetic diversity in AMF populations as it may be possible to breed and select more effective AMF for crop plants (Rodriguez and Sanders, 2015). Another related aspect that has to be considered is the fact that plant species, including crops, vary greatly in their responsiveness to AMF inoculation (Johnson et al., 1997; Smith and Smith, 2011; Smith et al., 2011). In modern agriculture, plant breeding programs, which result in varieties or cultivars with a range of genetic differences, should consider the plant response to AM fungi as a selection trait.

## Concluding remarks and perspectives

It is currently estimated that the world's population will exceed nine billion by 2050 (Rodriguez and Sanders, 2015). Thus, global agriculture will have to face the task of almost doubling food production but also of reducing the dependence of producers on agrochemicals (for the EU, see Directive 2009/128/EC regarding the sustainable use of pesticides), in order to safeguard human and environmental health. The forecasted necessary yield increase exceeds the current global capacity to produce food (Rodriguez and Sanders, 2015), thus highlighting the need to implement or revitalize eco-friendly technologies, such as AMF-based biofertilization. Despite its enormous potential, the application of AMF has not been fully adopted by farmers so far.

In this review, it has been pointed out that AMF inoculation overall produces positive outcomes on plant production in both controlled and open-field conditions, mainly due to the several nutrition-related benefits that this class of soil fungal symbionts is able to provide to their host-plant. In particular, AMF inoculation in the field has proven to be as effective as inoculation in the greenhouse, where non-inoculated controls are normally free of AMF, unlike in open-field conditions. For these reasons, the next significant step toward the stable use of AMF in agriculture is to carry out large-scale multi-location field trials and conduct cost-benefit analyses, such as that presented in Ceballos et al. (2013), in order to increase awareness among the potential end-users of the benefits of AMF inocula. In addition, since indigenous AMF have been demonstrated to be equally or even better performing than commercial or culture collection isolates, farmers are encouraged to autonomously produce their AMF inocula, starting from native soils. This makes the biofertilization technology more likely to be affordable for farmers, including those in developing countries who need their cropping system to be as highly sustainable as possible.

## Author contributions

AB, EL, RB, and VB contributed to the conception and drafting of the review. All the authors critically revised and approved the manuscript before submission.

### Conflict of interest statement

The authors declare that the research was conducted in the absence of any commercial or financial relationships that could be construed as a potential conflict of interest.

## Supplementary material

Supplementary Material S1 **The list of publications reviewed in Supplementary Material S2**.

Supplementary Material S2 **The table reports the authors of the study, the count of articles that group more than one experiment, the year of publication, the scientific journal, the cultivation condition in which the experiment was carried out (field, greenhouse, outdoor pot, or growth chamber), the stress type applied (abiotic, biotic, or none), the stress description, the list of host-plant species included in the experiment, the plant family they belong to, the origin of the inoculants (native, collection, commercial, non-native, or combined), the type of AMF propagule applied (spores, hyphae, or colonized roots), the application method (monospecies, multispecies or both), the tested AMF species list, the list of other microorganisms combined to the inocula tested in the experiment and the outcome of the inoculation process in terms of the presence/absence of a positive significant effect (sign. or non-sign., respectively) on root colonization, root and shoot biomass, yield, plant nutrition, and plant defense from a given pathogen**. As far as root colonization is concerned, any positive effect recorded in the experiment, regardless of whether it was found for the frequency, the intensity or the presence of arbuscules, was considered as significant. As for yield, any significant improvement, in terms of biomass or quality, was considered as significant. Plant defense from pathogen was considered as significant when plant survival, biomass and/or production were improved after AMF inoculation.

Supplementary Material S3 **The table reports the proportions of experiments showing a significant increase (sign./N) in colonization, biomass, yield, and nutrition following AMF inoculation for three factors, i.e., the experimental condition (levels = greenhouse or open-field), the inoculant origin (levels = native origin or other origin) and the method of application (levels = monospecies inoculum or multispecies inoculum)**. The number of experiments considered (N) for the calculation of each proportion is also reported.
