# Supplementary material for: Arbuscular Mycorrhizal Fungi as Natural Biofertilizers: Let's Benefit from Past Successes
Source: Front Microbiol. 2016 Jan 19;6:1559. doi: 10.3389/fmicb.2015.01559 (PMC4717633; doi:10.3389/fmicb.2015.01559)
Supplement: Supplementary Material S1 — The list of publications reviewed in Supplementary Material S2. [file DataSheet1.docx]

**List of publications reviewed in the Supplementary Material S2 table**

Affokpon, A., Coyne, D. L., Lawouin, L., Tossou, C., Agbèdè, R. D., and Coosemans, J. (2011). Effectiveness of native West African arbuscular mycorrhizal fungi in protecting vegetable crops against root-knot nematodes. *Biol. Fertil. Soils* 47, 207–217. doi:10.1007/s00374-010-0525-1.

Al-Karaki, G. N. (2002). Field Response of Garlic Inoculated with Arbuscular Mycorrhizal Fungi to Phosphorus Fertilization. *J. Plant Nutr.* 25, 747–756. doi:10.1081/PLN-120002956.

Alam, M., Khaliq, A., Sattar, A., Shukla, R. S., Anwar, M., and Dharni, S. (2011). Synergistic effect of arbuscular mycorrhizal fungi and *Bacillus subtilis* on the biomass and essential oil yield of rose-scented geranium ( *Pelargonium graveolens* ). *Arch. Agron. Soil Sci.* 57, 889–898. doi:10.1080/03650340.2010.498013.

Alguacil, M. M., Caravaca, F., and Roldan, A. (2005). Changes in rhizosphere microbial activity mediated by native or allochthonous AM fungi in the reafforestation of a Mediterranean degraded environment. *Biol. Fertil. Soils* 41, 59–68. doi:10.1007/s00374-004-0788-5.

Arias, M. S. B., Peña-Cabriales, J. J., Alarcón, A., and Maldonado Vega, M. (2015). Enhanced Pb Absorption by *Hordeum vulgare* L. and *Helianthus annuus* L. Plants Inoculated with an Arbuscular Mycorrhizal Fungi Consortium. *Int. J. Phytoremediation* 17, 405–413. doi:10.1080/15226514.2014.898023.

Aroca, R., del Mar Alguacil, M., Vernieri, P., and Ruiz-Lozano, J. M. (2008). Plant Responses to Drought Stress and Exogenous ABA Application are Modulated Differently by Mycorrhization in Tomato and an ABA-deficient Mutant (Sitiens). *Microb. Ecol.* 56, 704–719. doi:10.1007/s00248-008-9390-y.

Ashrafi, E., Zahedi, M., and Razmjoo, J. (2014). Co-inoculations of arbuscular mycorrhizal fungi and rhizobia under salinity in alfalfa. *Soil Sci. Plant Nutr.* 60, 619–629. doi:10.1080/00380768.2014.936037.

Asrar, A. A., Abdel-Fattah, G. M., and Elhindi, K. M. (2012). Improving growth, flower yield, and water relations of snapdragon (Antirhinum majus L.) plants grown under well-watered and water-stress conditions using arbuscular mycorrhizal fungi. *Photosynthetica* 50, 305–316. doi:10.1007/s11099-012-0024-8.

Badda, N., Yadav, K., Aggarwal, A., and Kadian, N. (2015). Consortium Effect of Arbuscular Mycorrhizal Fungi and *Pseudomonas fluorescens* with Various Levels of Superphosphate on Growth Improvement of Cotton ( *G. arboreum* L.). *J. Nat. Fibers* 12, 12–25. doi:10.1080/15440478.2013.879085.

Badda, N., Yadav, K., Kadian, N., and Aggarwal, A. (2013). Impact of Arbuscular Mycorrhizal Fungi with *Trichoderma viride* and *Pseudomonas fluorescens* on Growth Enhancement of Genetically Modified Bt Cotton ( *Bacillus* *thuringiensis* ). *J. Nat. Fibers* 10, 309–325. doi:10.1080/15440478.2013.791913.

Beltrano, J., Ruscitti, M., Arango, M. C., and Ronco, M. (2013). Effects of arbuscular mycorrhiza inoculation on plant growth, biological and physiological parameters and mineral nutrition in pepper grown under different salinity and p levels. *J. Soil Sci. Plant Nutr.* 13, 123–141.

Bhattacharya, S., and Bagyaraj, D. J. (2002). Effectiveness of Arbuscular Mycorrhizal Fungal Isolates on Arabica Coffee ( *Coffea arabica* L.). *Biol. Agric. Hortic.* 20, 125–131. doi:10.1080/01448765.2002.9754956.

Birhane, E., Kuyper, T. W., Sterck, F. J., Gebrehiwot, K., and Bongers, F. (2015). Arbuscular mycorrhiza and water and nutrient supply differently impact seedling performance of dry woodland species with different acquisition strategies. *Plant Ecol. Divers.* 8, 387–399. doi:10.1080/17550874.2014.992488.

Birhane, E., Sterck, F. J., Fetene, M., Bongers, F., and Kuyper, T. W. (2012). Arbuscular mycorrhizal fungi enhance photosynthesis, water use efficiency, and growth of frankincense seedlings under pulsed water availability conditions. *Oecologia* 169, 895–904. doi:10.1007/s00442-012-2258-3.

Briccoli Bati, C., Santilli, E., and Lombardo, L. (2015). Effect of arbuscular mycorrhizal fungi on growth and on micronutrient and macronutrient uptake and allocation in olive plantlets growing under high total Mn levels. *Mycorrhiza* 25, 97–108. doi:10.1007/s00572-014-0589-0.

Busquets, M., Calvet, C., Camprubí, A., and Estaún, V. (2010). Differential effects of two species of arbuscular mycorrhiza on the growth and water relations of Spartium junceum and Anthyllis cytisoides. *Symbiosis* 52, 95–101. doi:10.1007/s13199-010-0097-8.

Campanelli, A., Ruta, C., Tagarelli, A., Morone-Fortunato, I., and De Mastro, G. (2014). Effectiveness of mycorrhizal fungi on globe artichoke ( *Cynara cardunculus* L. var. *scolymus* ) micropropagation. *J. Plant Interact.* 9, 100–106. doi:10.1080/17429145.2013.770928.

Camprubí, A., Estaún, V., Nogales, A., García-Figueres, F., Pitet, M., and Calvet, C. (2008). Response of the grapevine rootstock Richter 110 to inoculation with native and selected arbuscular mycorrhizal fungi and growth performance in a replant vineyard. *Mycorrhiza* 18, 211–216. doi:10.1007/s00572-008-0168-3.

Carretero, C. L., Cantos, M., García, J. L., Azcón, R., and Troncoso, A. (2008). Arbuscular-Mycorrhizal Contributes to Alleviation of Salt Damage in Cassava Clones. *J. Plant Nutr.* 31, 959–971. doi:10.1080/01904160802043296.

Cartmill, A. D., Alarcón, A., and Valdez-Aguilar, L. A. (2007). Arbuscular Mycorrhizal Fungi Enhance Tolerance of *Rosa multiflora* cv. Burr to Bicarbonate in Irrigation Water. *J. Plant Nutr.* 30, 1517–1540. doi:10.1080/01904160701556802.

Ceballos, I., Ruiz, M., Fernández, C., Peña, R., Rodríguez, A., and Sanders, I. R. (2013). The In Vitro Mass-Produced Model Mycorrhizal Fungus, Rhizophagus irregularis, Significantly Increases Yields of the Globally Important Food Security Crop Cassava. *PLoS ONE* 8. doi:10.1371/journal.pone.0070633.

Conversa, G., Lazzizera, C., Bonasia, A., and Elia, A. (2013). Yield and phosphorus uptake of a processing tomato crop grown at different phosphorus levels in a calcareous soil as affected by mycorrhizal inoculation under field conditions. *Biol. Fertil. Soils* 49, 691–703. doi:10.1007/s00374-012-0757-3.

Copetta, A., Bardi, L., Bertolone, E., and Berta, G. (2011). Fruit production and quality of tomato plants ( *Solanum lycopersicum* L.) are affected by green compost and arbuscular mycorrhizal fungi. *Plant Biosyst. - Int. J. Deal. Asp. Plant Biol.* 145, 106–115. doi:10.1080/11263504.2010.539781.

Corkidi, L., Allen, E. B., Merhaut, D., Allen, M. F., Downer, J., Bohn, J., et al. (2004). Assessing the infectivity of commercial mycorrhizal inoculants in plant nursery conditions. *J. Environ. Hortic.* 22, 149–154.

Dare, M. O., Abaidoo, R. C., Fagbola, O., and Asiedu, R. (2010). Effects of Arbuscular Mycorrhizal Inoculation and Phosphorus Application on Yield and Nutrient Uptake of Yam. *Commun. Soil Sci. Plant Anal.* 41, 2729–2743. doi:10.1080/00103624.2010.518264.

Doubková, P., Kohout, P., and Sudová, R. (2013). Soil nutritional status, not inoculum identity, primarily determines the effect of arbuscular mycorrhizal fungi on the growth of Knautia arvensis plants. *Mycorrhiza* 23, 561–572. doi:10.1007/s00572-013-0494-y.

Douds, D. D., Nagahashi, G., Reider, C., and Hepperly, P. R. (2007). Inoculation with Arbuscular Mycorrhizal Fungi Increases the Yield of Potatoes in a High P Soil. *Biol. Agric. Hortic.* 25, 67–78. doi:10.1080/01448765.2007.10823209.

Dutt, S., Sharma, S. D., and Kumar, P. (2013). Inoculation of apricot seedlings with indigenous arbuscular mycorrhizal fungi in optimum phosphorus fertilization for quality growth attributes. *J. Plant Nutr.* 36, 15–31. doi:10.1080/01904167.2012.732648.

Estrada, B., Aroca, R., Azcón-Aguilar, C., Barea, J. M., and Ruiz-Lozano, J. M. (2013). Importance of native arbuscular mycorrhizal inoculation in the halophyte Asteriscus maritimus for successful establishment and growth under saline conditions. *Plant Soil* 370, 175–185. doi:10.1007/s11104-013-1635-y.

Farmer, M. J., Li, X., Feng, G., Zhao, B., Chatagnier, O., Gianinazzi, S., et al. (2007). Molecular monitoring of field-inoculated AMF to evaluate persistence in sweet potato crops in China. *Appl. Soil Ecol.* 35, 599–609. doi:10.1016/j.apsoil.2006.09.012.

Feng, G., Zhang, F., Li, X., Tian, C., Tang, C., and Rengel, Z. (2002). Uptake of nitrogen from indigenous soil pool by cotton plant inoculated with arbuscular mycorrhizal fungi. *Commun. Soil Sci. Plant Anal.* 33, 3825–3836. doi:10.1081/CSS-120015925.

Fini, A., Frangi, P., Amoroso, G., Piatti, R., Faoro, M., Bellasio, C., et al. (2011). Effect of controlled inoculation with specific mycorrhizal fungi from the urban environment on growth and physiology of containerized shade tree species growing under different water regimes. *Mycorrhiza* 21, 703–719. doi:10.1007/s00572-011-0370-6.

Gai, J. P., Feng, G., Christie, P., and Li, X. L. (2006). Screening of Arbuscular Mycorrhizal Fungi for Symbiotic Efficiency with Sweet Potato. *J. Plant Nutr.* 29, 1085–1094. doi:10.1080/01904160600689225.

Galván, G. A., Kuyper, T. W., Burger, K., Keizer, L. C. P., Hoekstra, R. F., Kik, C., et al. (2011). Genetic analysis of the interaction between *Allium* species and arbuscular mycorrhizal fungi. *Theor. Appl. Genet.* 122, 947–960. doi:10.1007/s00122-010-1501-8.

Garg, N., Manchanda, G., and Singla, P. (2014). Analysis of emergence stage facilitates the evaluation of chickpea (*Cicer arietinum* L.) genotypes for salinity tolerance imparted by mycorrhizal colonization. *Acta Physiol. Plant.* 36, 2651–2669. doi:10.1007/s11738-014-1636-3.

Garg, N., and Pandey, R. (2015). Effectiveness of native and exotic arbuscular mycorrhizal fungi on nutrient uptake and ion homeostasis in salt-stressed *Cajanus cajan* L. (Millsp.) genotypes. *Mycorrhiza* 25, 165–180. doi:10.1007/s00572-014-0600-9.

Garmendia, I., and Mangas, V. J. (2014). Comparative Study of Substrate-Based and Commercial Formulations of Arbuscular Mycorrhizal Fungi in Romaine Lettuce Subjected to Salt Stress. *J. Plant Nutr.* 37, 1717–1731. doi:10.1080/01904167.2014.889149.

Gaur, A., and Adholeya, A. (2002). Arbuscular-mycorrhizal inoculation of five tropical fodder crops and inoculum production in marginal soil amended with organic matter. *Biol. Fertil. Soils* 35, 214–218. doi:10.1007/s00374-002-0457-5.

Gaur, A., and Adholeya, A. (2005). Diverse Response of Five Ornamental Plant Species to Mixed Indigenous and Single Isolate Arbuscular-Mycorrhizal Inocula in Marginal Soil Amended with Organic Matter. *J. Plant Nutr.* 28, 707–723. doi:10.1081/PLN-200052647.

Gosling, P., Jones, J., and Bending, G. D. (2015). Evidence for functional redundancy in arbuscular mycorrhizal fungi and implications for agroecosystem management. *Mycorrhiza*, 1–7. doi:10.1007/s00572-015-0651-6.

Grümberg, B. C., Urcelay, C., Shroeder, M. A., Vargas-Gil, S., and Luna, C. M. (2015). The role of inoculum identity in drought stress mitigation by arbuscular mycorrhizal fungi in soybean. *Biol. Fertil. Soils* 51, 1–10. doi:10.1007/s00374-014-0942-7.

Gryndler, M., Vosátka, M., Hrŝelová, H., Catská, V., Chvátalová, I., and Jansa, J. (2002). Effect of dual inoculation with arbuscular mycorrhizal fungi and bacteria on growth and mineral nutrition of strawberry. *J. Plant Nutr.* 25, 1341–1358. doi:10.1081/PLN-120004393.

Guo, T., Zhang, J., Christie, P., and Li, X. (2007). Pungency of Spring Onion as Affected by Inoculation with Arbuscular Mycorrhizal Fungi and Sulfur Supply. *J. Plant Nutr.* 30, 1023–1034. doi:10.1080/01904160701394311.

Hart, M., Ehret, D. L., Krumbein, A., Leung, C., Murch, S., Turi, C., et al. (2015). Inoculation with arbuscular mycorrhizal fungi improves the nutritional value of tomatoes. *Mycorrhiza* 25, 359–376. doi:10.1007/s00572-014-0617-0.

Hashem, A., Abd_Allah, E. F., Alqarawi, A. A., Aldubise, A., and Egamberdieva, D. (2015). Arbuscular mycorrhizal fungi enhances salinity tolerance of *Panicum turgidum* Forssk by altering photosynthetic and antioxidant pathways. *J. Plant Interact.* 10, 230–242. doi:10.1080/17429145.2015.1052025.

Hayek, S., Grosch, R., Gianinazzi-Pearson, V., and Franken, P. (2012). Bioprotection and alternative fertilisation of petunia using mycorrhiza in a soilless production system. *Agron. Sustain. Dev.* 32, 765–771. doi:10.1007/s13593-012-0083-z.

Hernádi, I., Sasvári, Z., Albrechtová, J., Vosátka, M., and Posta, K. (2012). Arbuscular mycorrhizal inoculant increases yield of spice pepper and affects the indigenous fungal community in the field. *Hortscience* 47, 603–606.

Jha, A., Kumar, A., Saxena, R. K., Kamalvanshi, M., and Chakravarty, N. (2012). Effect of Arbuscular Mycorrhizal Inoculations on Seedling Growth and Biomass Productivity of Two Bamboo Species. *Indian J. Microbiol.* 52, 281–285. doi:10.1007/s12088-011-0213-3.

Jin, H., Germida, J. J., and Walley, F. L. (2013). Impact of arbuscular mycorrhizal fungal inoculants on subsequent arbuscular mycorrhizal fungi colonization in pot-cultured field pea (Pisum sativum L.). *Mycorrhiza* 23, 45–59. doi:10.1007/s00572-012-0448-9.

Kähkölä, A.-K., Nygren, P., Leblanc, H. A., Pennanen, T., and Pietikäinen, J. (2012). Leaf and root litter of a legume tree as nitrogen sources for cacaos with different root colonisation by arbuscular mycorrhizae. *Nutr. Cycl. Agroecosystems* 92, 51–65. doi:10.1007/s10705-011-9471-z.

Kapoor, R., Chaudhary, V., and Bhatnagar, A. K. (2007). Effects of arbuscular mycorrhiza and phosphorus application on artemisinin concentration in Artemisia annua L. *Mycorrhiza* 17, 581–587. doi:10.1007/s00572-007-0135-4.

Kapulnik, Y., Tsror (Lahkim), L., Zipori, I., Hazanovsky, M., Wininger, S., and Dag, A. (2010). Effect of AMF application on growth, productivity and susceptibility to Verticillium wilt of olives grown under desert conditions. *Symbiosis* 52, 103–111. doi:10.1007/s13199-010-0085-z.

Karthikeyan, A., and Muthukumar, T. (2006). Growth response of *Acacia planifrons* W. *et* a. to arbuscular mycorrhizal fungi and nitrogen fixing bacteria under nursery conditions. *For. Trees Livelihoods* 16, 269–275. doi:10.1080/14728028.2006.9752564.

Karthikeyan, A., Muthukumar, T., and Udaiyan, K. (2005). Response of Tea (*Camellia sinensis* (L). Kuntze) to Arbuscular Mycorrhizal Fungi under Plantation Nursery Conditions. *Biol. Agric. Hortic.* 22, 305–319. doi:10.1080/01448765.2005.9755294.

Karthikeyan, A., and Prakash, M. S. (2008). Effects of arbuscular mycorrhizal fungi, *Phosphobacterium* and *Azospirillum sp.* on the successful establishment of *Eucalyptus camaldulensis* Dehn. in bauxite mine spoils. *For. Trees Livelihoods* 18, 183–191. doi:10.1080/14728028.2008.9752628.

Kohler, J., Caravaca, F., del Mar Alguacil, M., and Roldán, A. (2009). Elevated CO_2_ increases the effect of an arbuscular mycorrhizal fungus and a plant-growth-promoting rhizobacterium on structural stability of a semiarid agricultural soil under drought conditions. *Soil Biol. Biochem.* 41, 1710–1716. doi:10.1016/j.soilbio.2009.05.014.

Kroeff Schmitz, J. A., Dutra de Souza, P. V., and Koller, O. C. (2001). Vegetative growth of *Poncirus trifoliata* L. Raf. inoculated with mycorrhizal fungi in three growing media. *Commun. Soil Sci. Plant Anal.* 32, 3031–3043. doi:10.1081/CSS-120001105.

Labidi, S., Jeddi, F. B., Tisserant, B., Yousfi, M., Sanaa, M., Dalpé, Y., et al. (2015). Field application of mycorrhizal bio-inoculants affects the mineral uptake of a forage legume (Hedysarum coronarium L.) on a highly calcareous soil. *Mycorrhiza* 25, 297–309. doi:10.1007/s00572-014-0609-0.

Latef, A. A. H. A. (2011). Influence of arbuscular mycorrhizal fungi and copper on growth, accumulation of osmolyte, mineral nutrition and antioxidant enzyme activity of pepper (*Capsicum annuum* L.). *Mycorrhiza* 21, 495–503. doi:10.1007/s00572-010-0360-0.

Lendenmann, M., Thonar, C., Barnard, R. L., Salmon, Y., Werner, R. A., Frossard, E., et al. (2011). Symbiont identity matters: carbon and phosphorus fluxes between *Medicago truncatula* and different arbuscular mycorrhizal fungi. *Mycorrhiza* 21, 689–702. doi:10.1007/s00572-011-0371-5.

Li, H., Wang, C., Li, X., and Xiang, D. (2013). Inoculating maize fields with earthworms (*Aporrectodea trapezoides*) and an arbuscular mycorrhizal fungus (*Rhizophagus intraradices*) improves mycorrhizal community structure and increases plant nutrient uptake. *Biol. Fertil. Soils* 49, 1167–1178. doi:10.1007/s00374-013-0815-5.

Li, H., Xiang, D., Wang, C., Li, X., and Lou, Y. (2012). Effects of epigeic earthworm (Eisenia fetida) and arbuscular mycorrhizal fungus (Glomus intraradices) on enzyme activities of a sterilized soil–sand mixture and nutrient uptake by maize. *Biol. Fertil. Soils* 48, 879–887. doi:10.1007/s00374-012-0679-0.

Li, M., Liu, R., Christie, P., and Li, X. (2005). Influence of Three Arbuscular Mycorrhizal Fungi and Phosphorus on Growth and Nutrient Status of Taro. *Commun. Soil Sci. Plant Anal.* 36, 2383–2396. doi:10.1080/00103620500253134.

Liu, A., and Dalpé, Y. (2009). Reduction in soil polycyclic aromatic hydrocarbons by arbuscular mycorrhizal leek plants. *Int. J. Phytoremediation* 11, 39–52. doi:10.1080/15226510802363444.

Maiti, D., Toppo, N. N., and Variar, M. (2011). Integration of crop rotation and arbuscular mycorrhiza (AM) inoculum application for enhancing AM activity to improve phosphorus nutrition and yield of upland rice (Oryza sativa L.). *Mycorrhiza* 21, 659–667. doi:10.1007/s00572-011-0376-0.

Malusa, E., Sas-Paszt, L., Popinska, W., and Zurawicz, E. (2007). The Effect of a Substrate Containing Arbuscular Mycorrhizal Fungi and Rhizosphere Microorganisms (Trichoderma, Bacillus, Pseudomonas and Streptomyces) and Foliar Fertilization on Growth Response and Rhizosphere pH of Three Strawberry Cultivars. *Int. J. Fruit Sci.* 6, 25–41. doi:10.1300/J492v06n04_04.

Mamatha, G., Bagyaraj, D., and Jaganath, S. (2002). Inoculation of field-established mulberry and papaya with arbuscular mycorrhizal fungi and a mycorrhiza helper bacterium. *Mycorrhiza* 12, 313–316. doi:10.1007/s00572-002-0200-y.

Mandal, S., Upadhyay, S., Wajid, S., Ram, M., Jain, D. C., Singh, V. P., et al. (2015). Arbuscular mycorrhiza increase artemisinin accumulation in Artemisia annua by higher expression of key biosynthesis genes via enhanced jasmonic acid levels. *Mycorrhiza* 25, 345–357. doi:10.1007/s00572-014-0614-3.

Mardukhi, B., Rejali, F., Daei, G., Ardakani, M. R., Malakouti, M. J., and Miransari, M. (2015). Mineral Uptake of Mycorrhizal Wheat ( *Triticum aestivum* L.) under Salinity Stress. *Commun. Soil Sci. Plant Anal.* 46, 343–357. doi:10.1080/00103624.2014.981271.

Mohammad, M. J., Malkawi, H. I., and Shibli, R. (2003). Effects of Arbuscular Mycorrhizal Fungi and Phosphorus Fertilization on Growth and Nutrient Uptake of Barley Grown on Soils with Different Levels of Salts. *J. Plant Nutr.* 26, 125–137. doi:10.1081/PLN-120016500.

Mortimer, P. E., Le Roux, M. R., Pérez-Fernández, M. A., Benedito, V. A., Kleinert, A., Xu, J., et al. (2013). The dual symbiosis between arbuscular mycorrhiza and nitrogen fixing bacteria benefits the growth and nutrition of the woody invasive legume *Acacia cyclops* under nutrient limiting conditions. *Plant Soil* 366, 229–241. doi:10.1007/s11104-012-1421-2.

Muthukumar, T., Udaiyan, K., and Rajeshkannan, V. (2001). Response of neem (Azadirachta indica A. Juss) to indigenous arbuscular mycorrhizal fungi, phosphate-solubilizing and asymbiotic nitrogen-fixing bacteria under tropical nursery conditions. *Biol. Fertil. Soils* 34, 417–426.

Ndoye, F., Kane, A., Bakhoum, N., Sanon, A., Fall, D., Diouf, D., et al. (2013). Response of *Acacia senegal* (L.) Willd. to inoculation with arbuscular mycorrhizal fungi isolates in sterilized and unsterilized soils in Senegal. *Agrofor. Syst.* 87, 941–952. doi:10.1007/s10457-013-9610-4.

Nell, M., Vötsch, M., Vierheilig, H., Steinkellner, S., Zitterl-Eglseer, K., Franz, C., et al. (2009). Effect of phosphorus uptake on growth and secondary metabolites of garden sage ( *Salvia officinalis* L.). *J. Sci. Food Agric.* 89, 1090–1096. doi:10.1002/jsfa.3561.

Olawuyi, O. J., Odebode, A. C., Olakojo, S. A., Popoola, O. O., Akanmu, A. O., and Izenegu, J. O. (2014). Host–pathogen interaction of maize ( *Zea mays* L.) and *Aspergillus niger* as influenced by arbuscular mycorrhizal fungi ( *Glomus deserticola* ). *Arch. Agron. Soil Sci.* 60, 1577–1591. doi:10.1080/03650340.2014.902533.

Oliveira, R. S., Vosátka, M., Dodd, J. C., and Castro, P. M. L. (2005). Studies on the diversity of arbuscular mycorrhizal fungi and the efficacy of two native isolates in a highly alkaline anthropogenic sediment. *Mycorrhiza* 16, 23–31. doi:10.1007/s00572-005-0010-0.

Ortas, I., Sari, N., Akpinar, Ç., and Yetisir, H. (2011). Screening mycorrhiza species for plant growth, P and Zn uptake in pepper seedling grown under greenhouse conditions. *Sci. Hortic.* 128, 92–98. doi:10.1016/j.scienta.2010.12.014.

Ouzounidou, G., Skiada, V., Papadopoulou, K. K., Stamatis, N., Kavvadias, V., Eleftheriadis, E., et al. (2015). Effects of soil pH and arbuscular mycorrhiza (AM) inoculation on growth and chemical composition of chia (Salvia hispanica L.) leaves. *Braz. J. Bot.* doi:10.1007/s40415-015-0166-6.

Patharajan, S., and Raaman, N. (2012). Influence of arbuscular mycorrhizal fungi on growth and selenium uptake by garlic plants. *Arch. Phytopathol. Plant Prot.* 45, 138–151. doi:10.1080/03235408.2010.501166.

Pellegrino, E., Turrini, A., Gamper, H. A., Cafà, G., Bonari, E., Young, J. P. W., et al. (2012). Establishment, persistence and effectiveness of arbuscular mycorrhizal fungal inoculants in the field revealed using molecular genetic tracing and measurement of yield components. *New Phytol.* 194, 810–822. doi:10.1111/j.1469-8137.2012.04090.x.

Peng, J., Li, Y., Shi, P., Chen, X., Lin, H., and Zhao, B. (2011). The differential behavior of arbuscular mycorrhizal fungi in interaction with Astragalus sinicus L. under salt stress. *Mycorrhiza* 21, 27–33. doi:10.1007/s00572-010-0311-9.

Perner, H., Schwarz, D., Bruns, C., Mäder, P., and George, E. (2007). Effect of arbuscular mycorrhizal colonization and two levels of compost supply on nutrient uptake and flowering of pelargonium plants. *Mycorrhiza* 17, 469–474. doi:10.1007/s00572-007-0116-7.

Prasad, A., Kumar, S., Khaliq, A., and Pandey, A. (2011). Heavy metals and arbuscular mycorrhizal (AM) fungi can alter the yield and chemical composition of volatile oil of sweet basil (Ocimum basilicum L.). *Biol. Fertil. Soils* 47, 853–861. doi:10.1007/s00374-011-0590-0.

Prasad, A., Kumar, S., Pandey, A., and Chand, S. (2012). Microbial and chemical sources of phosphorus supply modulate the yield and chemical composition of essential oil of rose-scented geranium (Pelargonium species) in sodic soils. *Biol. Fertil. Soils* 48, 117–122. doi:10.1007/s00374-011-0578-9.

Quatrini, P., Scaglione, G., Incannella, G., Badalucco, L., Puglia, A. M., and La Mantia, T. (2003). Microbial inoculants on woody legumes to recover a municipal landfill site. *Water Air Soil Pollut. Focus* 3, 189–199.

Quilambo, O., Weissenhorn, I., Doddema, H., Kuiper, P., and Stulen, I. (2005). Arbuscular Mycorrhizal Inoculation of Peanut in Low-Fertile Tropical Soil. II. Alleviation of Drought Stress. *J. Plant Nutr.* 28, 1645–1662. doi:10.1080/01904160500203606.

Ray, J. G., and Valsalakumar, N. (2010). Arbuscular mycorrhizal fungi and *Piriformospora indica* individually and in combination with *Rhizobium* on green gram. *J. Plant Nutr.* 33, 285–298. doi:10.1080/01904160903435409.

Robinson Boyer, L., Brain, P., Xu, X.-M., and Jeffries, P. (2015). Inoculation of drought-stressed strawberry with a mixed inoculum of two arbuscular mycorrhizal fungi: effects on population dynamics of fungal species in roots and consequential plant tolerance to water deficiency. *Mycorrhiza* 25, 215–227. doi:10.1007/s00572-014-0603-6.

Rydlová, J., Jelínková, M., Dušek, K., Dušková, E., Vosátka, M., and Püschel, D. (2015a). Arbuscular mycorrhiza differentially affects synthesis of essential oils in coriander and dill. *Mycorrhiza*. doi:10.1007/s00572-015-0652-5.

Rydlová, J., Sýkorová, Z., Slavíková, R., and Turis, P. (2015b). The importance of arbuscular mycorrhiza for Cyclamen purpurascens subsp. immaculatum endemic in Slovakia. *Mycorrhiza*. doi:10.1007/s00572-015-0634-7.

Sabannavar, S. J., and Lakshman, H. C. (2011). Synergistic Interactions among *Azotobacter, Pseudomonas*, and Arbuscular Mycorrhizal Fungi on Two Varieties of *Sesamum Indicum* L. *Commun. Soil Sci. Plant Anal.* 42, 2122–2133. doi:10.1080/00103624.2011.596241.

Saito, M., Oba, H., and kojima, T. (2011). Effect of nitrogen on the sporulation of arbuscular mycorrhizal fungi colonizing several gramineous plant species. *Soil Sci. Plant Nutr.* 57, 29–34. doi:10.1080/00380768.2010.541869.

Santos Lima, C., Campos, M. A. da S., and da Silva, F. S. B. (2015). Mycorrhizal Fungi (AMF) increase the content of biomolecules in leaves of Inga vera Willd. seedlings. *Symbiosis* 65, 117–123. doi:10.1007/s13199-015-0325-3.

Sato, T., Ezawa, T., Cheng, W., and Tawaraya, K. (2015). Release of acid phosphatase from extraradical hyphae of arbuscular mycorrhizal fungus *Rhizophagus clarus*. *Soil Sci. Plant Nutr.* 61, 269–274. doi:10.1080/00380768.2014.993298.

Schalamuk, S., Cabello, M. N., Chidichimo, H., and Golik, S. (2011). Effects of Inoculation with *Glomus mosseae* in Conventionally Tilled and Nontilled Soils with Different Levels of Nitrogen Fertilization on Wheat Growth, Arbuscular Mycorrhizal Colonization, and Nitrogen Nutrition. *Commun. Soil Sci. Plant Anal.* 42, 586–598. doi:10.1080/00103624.2011.546924.

Schwarz, D., Welter, S., George, E., Franken, P., Lehmann, K., Weckwerth, W., et al. (2011). Impact of arbuscular mycorrhizal fungi on the allergenic potential of tomato. *Mycorrhiza* 21, 341–349. doi:10.1007/s00572-010-0345-z.

Shen, H., Yang, H., and Guo, T. (2011). Influence of arbuscular mycorrhizal fungi and ammonium:nitrate ratios on growth and pungency of spring onion plants. *J. Plant Nutr.* 34, 743–752. doi:10.1080/01904167.2011.540689.

Singh, P. K., Singh, M., and Vyas, D. (2010). Biocontrol of Fusarium Wilt of Chickpea using Arbuscular Mycorrhizal Fungi and *Rhizobium leguminosorum* Biovar. *Caryologia* 63, 349–353. doi:10.1080/00087114.2010.10589745.

Singh, R. K., and Gogoi, P. (2012). Augmented growth of long pepper in response to arbuscular mycorrhizal inoculation. *J. For. Res.* 23, 339–344. doi:10.1007/s11676-012-0262-4.

Stevens, K. J., Wall, C. B., and Janssen, J. A. (2011). Effects of arbuscular mycorrhizal fungi on seedling growth and development of two wetland plants, Bidens frondosa L., and Eclipta prostrata (L.) L., grown under three levels of water availability. *Mycorrhiza* 21, 279–288. doi:10.1007/s00572-010-0334-2.

Stewart, L. I., Hamel, C., Hogue, R., and Moutoglis, P. (2005). Response of strawberry to inoculation with arbuscular mycorrhizal fungi under very high soil phosphorus conditions. *Mycorrhiza* 15, 612–619. doi:10.1007/s00572-005-0003-z.

Tavasolee, A., Aliasgharzad, N., Salehi, G. R., Mardi, M., Asgharzadeh, A., and Akbarivala, S. (2011). Effects of Co-Inoculation with Arbuscular Mycorrhizal Fungi and Rhizobia on Fungal Occupancy in Chickpea Root and Nodule Determined by Real-Time PCR. *Curr. Microbiol.* 63, 107–114. doi:10.1007/s00284-011-9951-z.

Tawaraya, K., Hirose, R., and Wagatsuma, T. (2012). Inoculation of arbuscular mycorrhizal fungi can substantially reduce phosphate fertilizer application to Allium fistulosum L. and achieve marketable yield under field condition. *Biol. Fertil. Soils* 48, 839–843. doi:10.1007/s00374-012-0669-2.

Taylor, A., Pereira, N., Thomas, B., Pink, D. A. C., Jones, J. E., and Bending, G. D. (2015). Growth and nutritional responses to arbuscular mycorrhizal fungi are dependent on onion genotype and fungal species. *Biol. Fertil. Soils*. doi:10.1007/s00374-015-1027-y.

Tong, Y., Gabriel-Neumann, E., Ngwene, B., Krumbein, A., Baldermann, S., Schreiner, M., et al. (2013). Effects of single and mixed inoculation with two arbuscular mycorrhizal fungi in two different levels of phosphorus supply on β-carotene concentrations in sweet potato (Ipomoea batatas L.) tubers. *Plant Soil* 372, 361–374. doi:10.1007/s11104-013-1708-y.

Udo, I. A., Uguru, M. I., and Ogbuji, R. O. (2013). Comparative efficacy of arbuscular mycorrhizal fungi in combination with bioformulated *Paecilomyces lilacinus* against *Meloidogyne incognita* on tomato in two Ultisols of South-eastern Nigeria. *Biocontrol Sci. Technol.* 23, 1083–1097. doi:10.1080/09583157.2013.820254.

Urgiles, N., Strauß, A., Loján, P., and Schüßler, A. (2014). Cultured arbuscular mycorrhizal fungi and native soil inocula improve seedling development of two pioneer trees in the Andean region. *New For.* 45, 859–874. doi:10.1007/s11056-014-9442-8.

Velivelli, S. L. S., Kromann, P., Lojan, P., Rojas, M., Franco, J., Suarez, J. P., et al. (2015). Identification of mVOCs from Andean Rhizobacteria and Field Evaluation of Bacterial and Mycorrhizal Inoculants on Growth of Potato in its Center of Origin. *Microb. Ecol.* 69, 652–667. doi:10.1007/s00248-014-0514-2.

Vicente-Sánchez, J., Nicolás, E., Pedrero, F., Alarcón, J. J., Maestre-Valero, J. F., and Fernández, F. (2014). Arbuscular mycorrhizal symbiosis alleviates detrimental effects of saline reclaimed water in lettuce plants. *Mycorrhiza* 24, 339–348. doi:10.1007/s00572-013-0542-7.

Wang, C., Li, X., and Song, F. (2012). Protecting Cucumber from *Fusarium* Wilt with Arbuscular Mycorrhizal Fungi. *Commun. Soil Sci. Plant Anal.* 43, 2851–2864. doi:10.1080/00103624.2012.728263.

Wang, C., Li, X., Zhou, J., Wang, G., and Dong, Y. (2008a). Effects of Arbuscular Mycorrhizal Fungi on Growth and Yield of Cucumber Plants. *Commun. Soil Sci. Plant Anal.* 39, 499–509. doi:10.1080/00103620701826738.

Wang, M., Christie, P., Xiao, Z., Qin, C., Wang, P., Liu, J., et al. (2008b). Arbuscular mycorrhizal enhancement of iron concentration by Poncirus trifoliata L. Raf and Citrus reticulata Blanco grown on sand medium under different pH. *Biol. Fertil. Soils* 45, 65–72. doi:10.1007/s00374-008-0290-6.

Wang, X., Pan, Q., Chen, F., Yan, X., and Liao, H. (2011). Effects of co-inoculation with arbuscular mycorrhizal fungi and rhizobia on soybean growth as related to root architecture and availability of N and P. *Mycorrhiza* 21, 173–181. doi:10.1007/s00572-010-0319-1.

Williams, A., Norton, D., and Ridgway, H. (2012). Different arbuscular mycorrhizal inoculants affect the growth and survival of *Podocarpus cunninghamii* restoration plantings in the Mackenzie Basin, New Zealand. *N. Z. J. Bot.* 50, 473–479. doi:10.1080/0028825X.2012.672429.

Wu, Q.-S., Zou, Y.-N., and Wang, G.-Y. (2011). Arbuscular Mycorrhizal Fungi and Acclimatization of Micropropagated Citrus. *Commun. Soil Sci. Plant Anal.* 42, 1825–1832. doi:10.1080/00103624.2011.587570.

Xu, P., Liang, L. Z., Dong, X. Y., and Shen, R. F. (2015). Effect of arbuscular mycorrhizal fungi on aggregate stability of a clay soil inoculating with two different host plants. *Acta Agric. Scand. Sect. B — Soil Plant Sci.* 65, 23–29. doi:10.1080/09064710.2014.960887.

Yadav, A., Suri, V. K., Kumar, A., Choudhary, A. K., and Meena, A. L. (2015a). Enhancing Plant Water Relations, Quality, and Productivity of Pea ( *Pisum sativum* L.) through Arbuscular Mycorrhizal Fungi, Inorganic Phosphorus, and Irrigation Regimes in an Himalayan Acid Alfisol. *Commun. Soil Sci. Plant Anal.* 46, 80–93. doi:10.1080/00103624.2014.956888.

Yadav, A., Yadav, K., and Aggarwal, A. (2015b). Impact of Arbuscular Mycorrhizal Fungi with *Trichoderma viride* and *Pseudomonas fluorescens* on Growth, Yield and Oil Content in *Helianthus annuus* L. *J. Essent. Oil Bear. Plants* 18, 444–454. doi:10.1080/0972060X.2014.971066.

Yooyongwech, S., Phaukinsang, N., Cha-um, S., and Supaibulwatana, K. (2013). Arbuscular mycorrhiza improved growth performance in Macadamia tetraphylla L. grown under water deficit stress involves soluble sugar and proline accumulation. *Plant Growth Regul.* 69, 285–293. doi:10.1007/s10725-012-9771-6.

Yu, Y., Zhang, S., Huang, H., and Wu, N. (2010). Uptake of Arsenic by Maize Inoculated with Three Different Arbuscular Mycorrhizal Fungi. *Commun. Soil Sci. Plant Anal.* 41, 735–743. doi:10.1080/00103620903563964.

Zarei, M., and Paymaneh, Z. (2014). Effect of salinity and arbuscular mycorrhizal fungi on growth and some physiological parameters of *Citrus jambheri*. *Arch. Agron. Soil Sci.* 60, 993–1004. doi:10.1080/03650340.2013.853289.

Zarei, M., Saleh-Rastin, N., Alikhani, H. A., and Aliasgharzadeh, N. (2006). Responses of Lentil to Co-Inoculation with Phosphate-Solubilizing Rhizobial Strains and Arbuscular Mycorrhizal Fungi. *J. Plant Nutr.* 29, 1509–1522. doi:10.1080/01904160600837667.

Zhang, X., Chen, B., and Ohtomo, R. (2015). Mycorrhizal effects on growth, P uptake and Cd tolerance of the host plant vary among different AM fungal species. *Soil Sci. Plant Nutr.* 61, 359–368. doi:10.1080/00380768.2014.985578.

Zhang, Y., Yao, Q., Li, J., Hu, Y., and Chen, J. (2014). Growth Response and Nutrient Uptake of Eriobotrya japonica Plants Inoculated with Three Isolates of Arbuscular Mycorrhizal Fungi Under Water Stress Condition. *J. Plant Nutr.* 37, 690–703. doi:10.1080/01904167.2013.868478.

Zubek, S., Mielcarek, S., and Turnau, K. (2012). Hypericin and pseudohypericin concentrations of a valuable medicinal plant Hypericum perforatum L. are enhanced by arbuscular mycorrhizal fungi. *Mycorrhiza* 22, 149–156. doi:10.1007/s00572-011-0391-1.

Zubek, S., Stojakowska, A., Anielska, T., and Turnau, K. (2010). Arbuscular mycorrhizal fungi alter thymol derivative contents of Inula ensifolia L. *Mycorrhiza* 20, 497–504. doi:10.1007/s00572-010-0306-6.

Zubek, S., Turnau, K., Tsimilli-Michael, M., and Strasser, R. J. (2009). Response of endangered plant species to inoculation with arbuscular mycorrhizal fungi and soil bacteria. *Mycorrhiza* 19, 113–123. doi:10.1007/s00572-008-0209-y.
